# Parental High-Fat Diet Promotes Inflammatory and Senescence-Related Changes in Prostate

**DOI:** 10.1155/2017/4962950

**Published:** 2017-02-05

**Authors:** Kulbhushan Tikoo, Ajit Vikram, Shweta Shrivastava, Gopabandhu Jena, Heta Shah, Richa Chhabra

**Affiliations:** ^1^Laboratory of Epigenetics and Diseases, Department of Pharmacology and Toxicology, National Institute of Pharmaceutical Education and Research (NIPER), Mohali, Punjab 160 062, India; ^2^Facility for Risk Assessment and Intervention Studies, Department of Pharmacology and Toxicology, National Institute of Pharmaceutical Education and Research (NIPER), Mohali, Punjab 160 062, India

## Abstract

*Background*. Obesity and dietary habits are associated with increased incidences of aging-related prostatic diseases. The present study was aimed to investigate transgenerational effects of chronic high-fat diet (HFD) feeding on inflammation and senescence-related changes in prostate.* Methods*. Sprague-Dawley rats were kept on either normal or HFD one. Senescence-associated *β*-galactosidase (SA *β*-gal) activity, inflammation, and cellular proliferation were determined in the prostate.* Results*. Increased SA *β*-gal activity, expression of p53, and cell proliferation marker PCNA were observed in ventral prostate of HFD-fed rats. Immunostaining for p53 and PCNA revealed that the p53 immunopositive cells were primarily in stroma while PCNA immunopositive cells were epithelial cells. An increase in expression of cycloxygenase-2 (COX-2) and phosphorylation of nuclear factor-kappa B (NF-kB) was observed in prostate of weaning pups HFD-fed parents. However, in adult pups, irrespective of dietary habit, a significant increase in the expression of COX-2, PCNA, phosphorylation of NF-kB, infiltration of inflammatory cells, and SA *β*-gal activity was observed.* Conclusions.* Present investigation reports that HFD feeding promotes accumulation of p53 expressing cells, proliferation of epithelial cells, and senescence-related changes in prostate. Further, parental HFD-feeding upholds inflammatory, proliferative, and senescence-related changes in prostate of pups.

## 1. Introduction

Obesity has crossed the epidemic proportion in several countries, leading to an explosion of obesity-related health disorders. Genetic predispositions, western pattern (fat-rich) diet and sedentary lifestyle are the major contributors to increased prevalence of obesity. Despite the association between obesity and increased risk for cardiovascular diseases, type 2 diabetes and some cancers are well known; its link with prostatic diseases has been underappreciated. Aging is known as a single most important factor for the increased prevalence of prostatic disorders such as prostatic hyperplasia and prostate cancer [1, 2]. Recent reports indicating high incidences of age-related prostatic disorders in the obese and insulin-resistant individuals [3] have invigorated research interests to investigate the interrelation between these pathological conditions. Studies using animal models also largely support the association between metabolic syndrome and prostatic diseases [4–6]. Aging, sedentary lifestyle, and diet rich in fat are probably the most important factors for the high incidence of metabolic diseases, diabetes, and prostatic disorders. Although previous studies from our research group provide HFD-induced secondary hyperinsulinaemia as a partial explanation for the increased cell proliferation and prostatic enlargement in the rats [6–8], a precise understanding still remains unknown. The experimental as well as clinical studies demonstrate that prostatic health is greatly affected by dietary habits [4–6, 9–11]. Interestingly, higher incidences of prostatic dysplasia and prostatitis were observed in the pups exposed to the intrauterine maternal protein malnutrition [12]. Accumulating reports suggest association between offspring's health and the diet of either of the parents [13, 14].

To explain the higher susceptibility of obese and insulin-resistant men to the age-related prostatic diseases, we hypothesized that, apart from indirect growth promoting effects of dietary fats by inducing compensatory hyperinsulinaemia, excessive fat intake accelerates the prostatic aging and thus the incidence of age-related prostatic disorders. Further, the possibility of transgenerational influence of the dietary habit of parents on the prostate of pups at early as well as later stage of life was examined.

## 2. Materials and Methods

### 2.1. Animal Experiments

All the animal experiments were approved by the Institutional Animal Ethics Committee (IAEC) and were used according to the CPCSEA (Committee for the Purpose of Control and Supervision of Experimentation on Animals) guidelines. The Sprague-Dawley rats (4 weeks old) were procured from Institute's Central Animal Facility and were kept at controlled environmental conditions with room temperature (22 ± 2°C) and humidity (50 ± 10%). The 12 h light (0600–1800 h) and dark cycle was maintained throughout the study. Rats were allowed to access the food and water ad libitum and acclimatized for one week prior to the start of experiment. To investigate the effect of excessive dietary fat on the prostate, the first objective of the study, animals were kept on normal pellet diet (NPD) or a HFD (see the following for composition) for 25 to 28 weeks. At the time of sacrifice these rats were 30 to 33 weeks old. To investigate the transgenerational effects of dietary fat, the second objective of the study, HFD- or NPD-fed males and females were kept together (one male with three females) for one week (7th to 8th week after dietary manipulation). The plasma glucose and insulin levels were determined at the time gestation as well as lactation. To determine the biochemical parameters blood samples were collected from females one week after the males were separated from the females (9th week) and one week after the birth of the pups. The prostates of the pups of HFD-fed parents and NPD-fed parents were carefully isolated at the age of 4 weeks (weaning) and 16 weeks (adult). Further, the pups of the HFD-fed parents were subdivided into two groups and one group continued on HFD and other on NPD (H-NPD). Considering the previous studies where we examined proliferative characteristics of the HFD-fed pups of NPD-fed parents [6], the pups of NPD parents were not subdivided and kept on either NPD or HFD. All the animals were killed by cervical dislocation. Rodent prostate consists of bilaterally symmetrical ventral, dorsolateral, and anterior prostate. In the present investigation ventral prostate (VP) was used for the subsequent analysis. The left lobe of the VP was preserved at −80°C and used for the molecular studies. However, the right lobe of the VP was divided into three parts relative to the urethra (distal, intermediate, and proximal) and intermediate part was used for the immunohistochemical examination. In case of weaning pups entire prostate was used for the western blot analysis owing to smaller size of the prostate (15–30 mg). At the time of sacrifice epididymal fat pad (white adipose tissue, WAT) and interscapular fat (brown adipose tissue (BAT)) were carefully isolated and sum of the weight of WAT and BAT per 100 g of body weight was used as an indicator of obesity (adiposity index).

### 2.2. Diet for the Development of Experimental Insulin Resistance

Insulin resistance and obesity were induced in rats by feeding HFD (5.3 kcal/g, carbohydrate 17%, protein 25%, and fat 58% kcal), while the controls were fed with NPD (3.8 kcal/g, carbohydrate 67%, protein 21%, and fat 12% kcal). The NPD used to feed the animal was standard rodent chow (Pranav Agro Industries, New Delhi, India). The content of HFD includes NPD powder (36.5%), lard (31%), casein (25%), vitamin-mineral mix powder (6%), cholesterol (1%), DL-methionine (0.3%), Yee-sac powder (0.1%), and sodium chloride (0.1%). The detailed methodology for HFD preparation has already been described elsewhere [15]. The suitability of diet in inducing features of insulin resistance has been previously determined [6–8].

### 2.3. Assay of Biochemical Parameters

The blood samples (≈0.8 mL) were collected from the orbital plexus of rats under anaesthesia in heparinised microcentrifuge tubes. The plasma was separated by centrifugation and analyzed for glucose, triglycerides, total-cholesterol, and HDL-cholesterol using commercially available spectrophotometric kits (Accurex Biomedical Pvt. Ltd., India). Plasma insulin was estimated by rat/mouse insulin ELISA kit (rat/mouse insulin ELISA kit, Linco Research, USA) as per the manufacturer's instructions.

### 2.4. Glucose Tolerance Tests

Animals were kept on 6 h fasting and a basal sample was taken, followed by intraperitoneal injection of D-glucose (1,000 mg/kg, Sigma-Aldrich, USA). Blood samples were collected at 15, 30, 60, 90, and 120 min and plasma glucose concentration was determined to assess impairment in the glucose tolerance.

### 2.5. Determination of Senescence-Associated *β*-Galactosidase (SA *β*-gal) Activity

SA *β*-gal activity was examined in VP as described [16]. Briefly, the freshly isolated tissues were fixed in 3% formaldehyde, washed, and incubated for 2 hours at 37°C in *β*-gal staining solution containing 1 mg/mL 5-bromo-4-chloro-3-indolyl *β*-D-galactopyranoside (X-gal), 5 mmol/l potassium ferrocyanide, 5 mmol/l potassium ferricyanide, 150 mmol/l NaCl, 2 mmol/l MgCl_2_, 0.01% sodium deoxycholate, and 0.02% Nonidet-40, and 40 mM citric acid/sodium phosphate (pH 6.0). The senescence-associated *β*-galactosidase stained VPs were photographed, and RGB (red, green, and blue) colour intensity per unit area of image was quantitatively analyzed using MATLAB (Version 2011a) software. The relative intensity of red, green, and blue (RGB) colour was individually determined. As the X-gal staining led to the development of greenish-blue colour, the sum of these two colours was considered as a measure of the SA *β*-gal activity.

### 2.6. Histological and Immunohistochemical Examinations

Rats were anesthetized with diethyl ether and killed by cervical dislocation. VPs (distal, intermediate, and proximal) were stored in 10% formal saline. Paraffin blocks were prepared after completing the routine processing. The intermediate portions of VPs were used in histological processing. Prostatic histology was evaluated under hematoxylin and eosin (H&E) staining. For immunohistochemical examinations 3–5 *μ*m thick sections originating from intermediate part of the VP (right lobe) were prepared from paraffin blocks. Antigens were retrieved by heating (95°C, 20 minutes) in citrate buffer (10 mM). p53 and PCNA rabbit polyclonal primary antibodies (Santa Cruz Biotechnology, USA) were used in the study. Polyvalent biotinylated goat anti-rabbit secondary antibody and streptavidin peroxidase (STV-HRP) system was used to amplify the signals, followed by detection with diaminobenzidine (DAB) as a chromogen. Slides were counterstained with hematoxylin, dehydrated with alcohols and xylene, and mounted in DPX. To identify the compartmental change in the p53 and PCNA, the sections were first immunostained for p53 antibodies, images were captured, and then same slides were subjected to the immunostaining for PCNA. Reimmunostaining of prostatic sections with PCNA did not lead to any appreciable change in the frequency of PCNA positive cells (see Fig. S1 in the Supplementary Material available online at https://doi.org/10.1155/2017/4962950). However, a decrease in the intensity of first immunostaining (p53) was observed. Therefore the slides were subjected to immunostaining for PCNA after determining the frequency of p53 positive cells. Images were captured by charged coupled device (CCD) camera attached with the Olympus microscope (Model BX 51).

### 2.7. Immunoblotting and Immunoprecipitation

Protein samples were resolved on 10–12% SDS-PAGE, transferred to PVDF/nitrocellulose membrane, and analyzed with antibodies against PCNA (1 : 1000, rabbit), p53 (1 : 500, rabbit, wild-type), p38 (1 : 1000, rabbit), NF-kB (1 : 500, rabbit), p-NF-kB (1 : 500, rabbit), cyclooxygenase-2 (COX-2, 1 : 1000, rabbit), and *β*-actin (1 : 1000, rabbit). The primary and secondary antibodies were procured from Santa Cruz Biotechnology, Inc. USA. The antigen-primary antibody complexes were incubated with horseradish-peroxidase (HRP) conjugated secondary antibodies and visualized by western blotting luminol reagent (Santa Cruz Biotechnology, Inc., USA). Image was captured by ImageQuant-350 (Ver. 1.0.2). The protein quantification was done with ImageQuant TL (GE Healthcare, UK) software and intensity values were normalized to *β*-actin. Tissue lysate was precleaned by incubating with irrelevant primary antibody and protein A/G plus-Agarose for 90 minutes. The pellet obtained after centrifugation was discarded and the supernatant was used for the further processing. Precleaned tissue lysates (500 *μ*g protein) were incubated with p53/PCNA antibody for 16 h at 4°C. Subsequently, the immune complex was precipitated with Protein A/G plus-Agarose (Santa Cruz Biotechnology) for 6 h at 4°C. The immunoprecipitate was washed thrice with lysis buffer. The protein sample was resolved on SDS-PAGE and immunoblotted as described above.

### 2.8. Statistical Analysis

Statistical analysis was performed using SPSS (Version 17.0) statistical software. Significance of difference between two groups was evaluated using *t*-test. For multiple comparisons, one-way ANOVA was used and post hoc analysis was performed with Tukey's test. Results were considered significant if *P* values were ≤0.05.

## 3. Results

### 3.1. Effect of Chronic HFD Feeding on SA *β*-gal Activity

**Figure 1:**
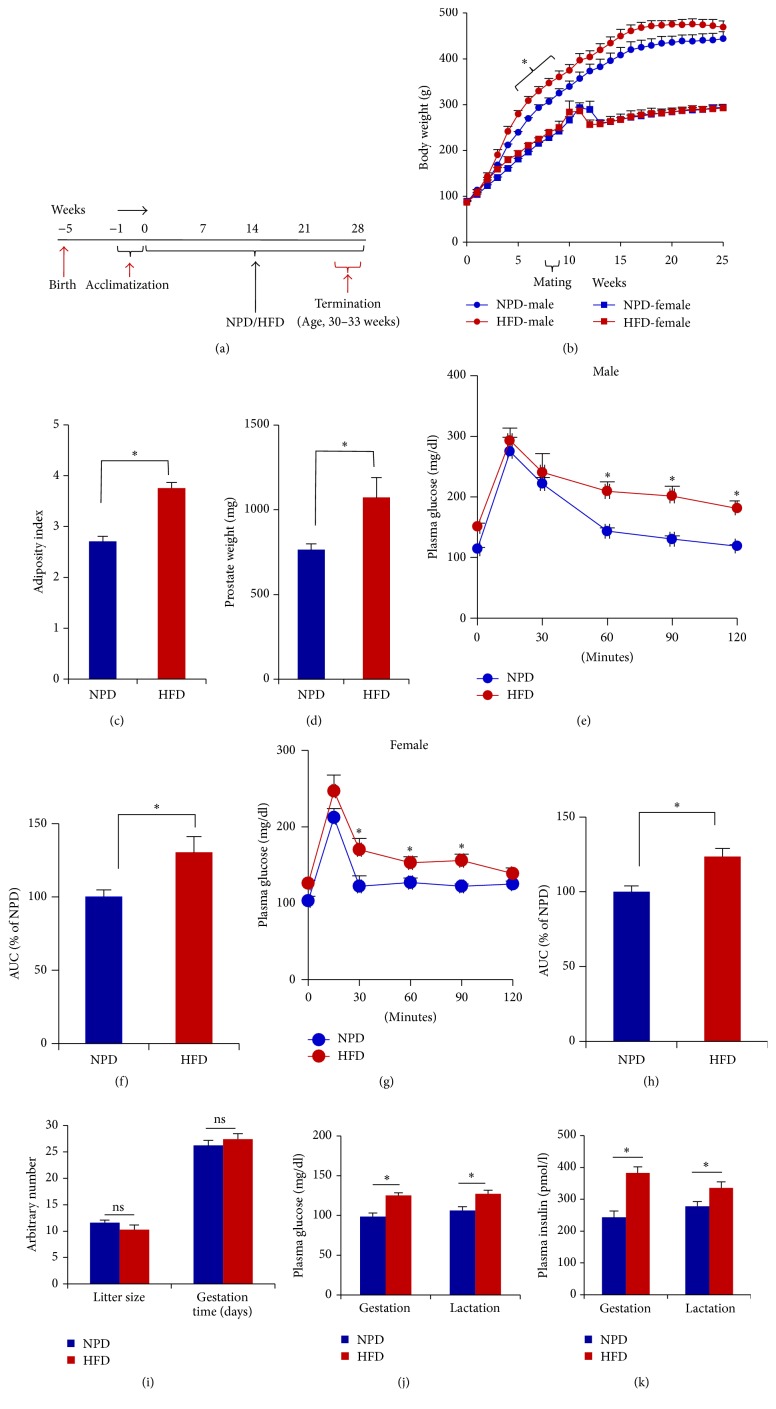
Chronic high-fat diet (HFD) feeding induces insulin resistance and prostatic enlargement. (a) Schematic diagram illustrating the experimental design. (b) Effect of chronic HFD feeding on the body weight of male and female rats (parents). (c) HFD feeding led to significant increase in the adiposity index in HFD-fed male rats as compared to age-matched NPD-fed control rats. (d) Effect of chronic HFD feeding on the ventral prostate (VP) weight. (e) Effect of chronic HFD feeding on the glucose tolerance in male rats. (f) Area under curve (AUC) of plasma glucose concentration and time curve (e). (g) The HFD-fed females were glucose intolerant. The glucose tolerance test was performed one week prior to keeping them for mating. (h) AUC of plasma glucose concentration and time curve (g). (i) No difference in the litter size and average gestation time was observed between HFD-fed and NPD-fed dams. (j and k) The plasma glucose and insulin levels were significantly higher in the HFD-fed females during gestation as well as lactation. The plasma glucose and insulin levels were determined one week after the males were separated and one week after the delivery of pups. All the values are shown as mean ± SEM. ^*∗*^*P* < 0.05 versus indicated group.

**Figure 2:**
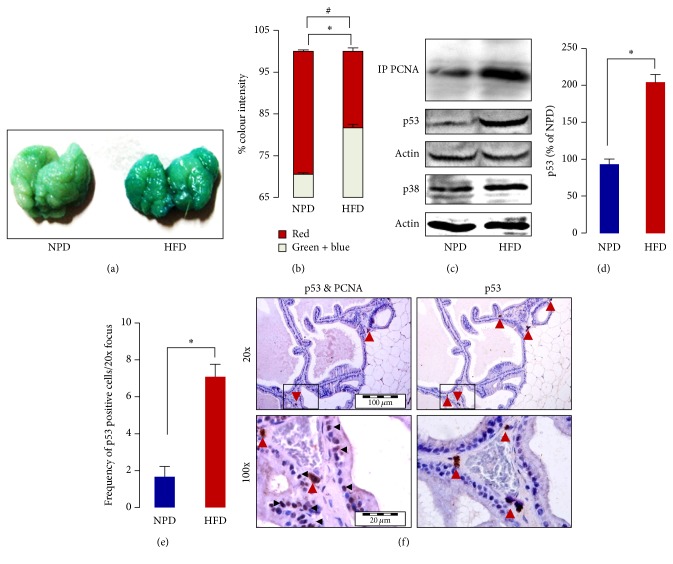
Chronic HFD feeding accelerates prostatic aging and cell proliferation. (a) Effect of chronic HFD feeding on the SA *β*-gal activity in VP. (b) A significant increase in the intensity of the sum of green and blue colour and decrease in the intensity of red colour were observed in the X-gal stained images of VP of HFD-fed rats. Statistical comparisons are, respectively, indicated by *∗* (green and blue) and # (red) symbol. (c) Chronic HFD feeding led to increased p38, p53, and PCNA expression in the VP. (d) A significant increase in the p53 expression was observed in the VP of HFD-fed rats as compared to the age-matched NPD-fed rats. (e) A significant increase in the frequency of p53 positive cells was observed in the VP of HFD-fed rats as compared to the age-matched NPD-fed rats. (f) Cellular localization of p53 and PCNA positive cells (magnification of 20x and 100x). Representative photomicrographs showing the immunohistochemical localization of p53 alone and p53 and PCNA in the prostatic sections (same slides were used for the observation). p53 positive cells were found to be primarily restricted to the stromal cells (indicated by red arrows) while those of PCNA (indicated by black arrows) were to the luminal epithelial cells. All the values are shown as mean ± SEM. ^*∗*^*P* < 0.05, ^#^*P* < 0.05 versus indicated group.

The rats were kept on HFD resulting in the obesity and insulin resistance as evidenced by increased body weight, adiposity, and impaired glucose tolerance (Figures 1(a)–1(c) and 1(e)–1(h)). A significant increase in the absolute and relative (data not shown) weight of ventral prostate (VP) was observed in the HFD-fed rats as compared to the age-matched NPD-fed control (Figure 1(d)). VP of HFD-fed rats showed enhanced activity of SA *β*-gal as compared to that of age-matched NPD-fed control (Figure 2(a)), which is indicative of higher lysosomal mass during replicative aging. Further, the image of SA *β*-gal stained VPs was analyzed for the RGB colour intensity (Fig. S2). A significant increase in the intensity of the sum of green and blue colour and decrease in the intensity of red colour were observed in the HFD-fed rats as compared to the age-matched NPD-fed controls (Figure 2(b)).

### 3.2. Effect of Chronic HFD Feeding on p53 and PCNA Expression in Prostate

**Figure 3:**
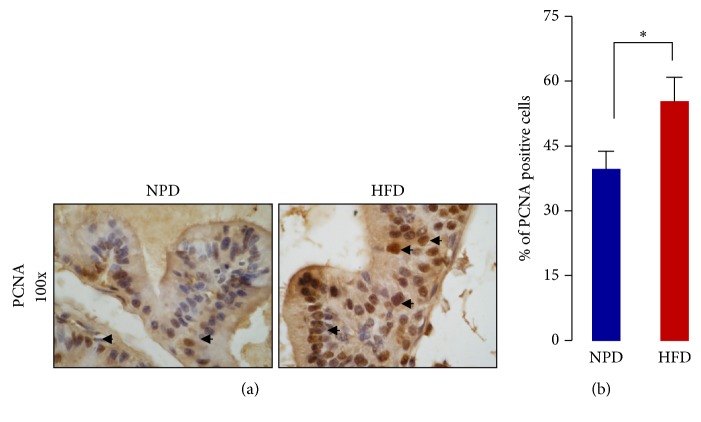
Chronic HFD feeding increases frequency of PCNA positive cells in VP.** (**a) Representative photomicrographs showing PCNA (magnification ×100). (b) A significant increase in the frequency of PCNA positive cells was observed in the VP of HFD-fed rats as compared to the age-matched NPD-fed rats. All the values are shown as mean ± SEM. ^*∗*^*P* < 0.05 versus indicated group.

The HFD-fed rats have shown increased prostatic expression of p53 and PCNA as compared to age-matched NPD-fed control. The increased expression of p53 and PCNA was further confirmed by immunohistochemical analysis of the prostatic section, and a significant increase in the frequency of p53 and PCNA positive cells was observed in the VP of chronic HFD-fed rats (Figures 2(c)–2(f), 3(a), and 3(b)). Importantly, most of the p53 positive cells were stromal cells, while PCNA expressing cells were primarily epithelial cells (Figure 2(f)).

### 3.3. Effect of Parental HFD Feeding on the Prostate

#### 3.3.1. Weaning Pups

**Figure 4:**
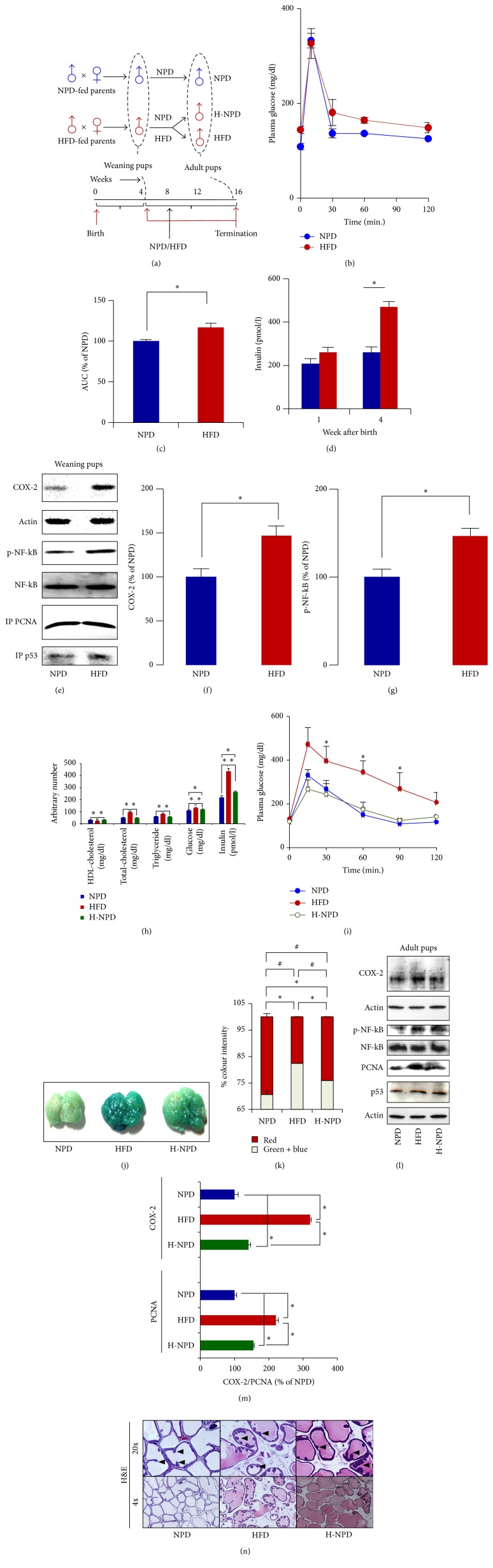
Parental HFD feeding promotes inflammation, proliferative, and senescence-related changes in the prostate. (a) Schematic diagram illustrating the experimental design. (b) The weaning male pups of HFD-fed parents were slightly glucose intolerant as compared to that of the NPD-fed parents. (c) AUC of plasma glucose concentration and time curve in (b). (d) An increase in the plasma insulin level in the pups of HFD-fed parents was observed at one week and 4 weeks (weaning) of age as compared to that of NPD-fed parents. (e) Increased expression of p53 and COX-2 and activation of NF-kB were observed in prostate of the weaning pups of HFD-fed parents as compared to the prostates of the pups of NPD-fed parents. However, no appreciable change in the PCNA expression was observed. (f) A significant increase in the COX-2 expression was observed in the VP of the pups of HFD-fed parents as compared to the pups of NPD-fed parents. (g) A significant increase in the phosphorylation of NF-kB was observed in the VP of the pups of HFD-fed parents as compared to the pups of NPD-fed parents. (h) The plasma total-cholesterol, triglyceride, glucose, and insulin levels were increased and HDL-cholesterol level decreased in the HFD-fed adult pups (16 weeks) of HFD-fed parents as compared to NPD-fed pups of NPD-fed parents. The plasma glucose and insulin levels of NPD-fed pups of HFD-fed parents were higher as compared to NPD-fed pups of NPD-fed parents. (i) The HFD-fed adult pups (16 weeks) of the HFD-fed parents were glucose intolerant, but improved glucose tolerance was observed in the NPD-fed pups of the HFD-fed parent rats. (j) The SA *β*-gal activity was found to be highest in the HFD-fed pups of the HFD-fed parents, followed by NPD-fed pups of the HFD-fed parents and NPD-fed pups of the NPD-fed parents. (k) A significant increase in the sum of the green and blue colour intensity was observed in the prostatic images of HFD-fed/NPD-fed pups of HFD-fed parents as compared to NPD-fed pups of NPD-fed parents (statistical comparisons are indicated by *∗* symbol). A significant decrease in the red colour intensity was observed in the prostatic images of HFD-fed/NPD-fed pups of HFD-fed parents as compared to NPD-fed pups of NPD-fed parents (statistical comparisons are indicated by # symbol). (l and m) Increased expression of p53, PCNA, COX-2, and activation of p-NF-kB was observed in the prostate of adult HFD-fed/NPD-fed pups of HFD-fed parents as compared to that of the age-matched NPD-fed pups of the NPD-fed parents. (n) Representative photomicrographs showing increased epithelial layer infolding (arrow heads) in the prostatic sections of HFD and NPD-fed adult pups of HFD-fed parents as compared to NPD-fed pups of NPD-fed parents. All the values are shown as mean ± SEM. ^*∗*^*P* < 0.05, ^#^*P* < 0.05 versus indicated group.

The HFD-fed female rats were glucose intolerant and had significantly higher plasma glucose and insulin level during gestation as well as lactation as compared to the NPD controls. There was no significant difference in the litter size, male to female ratio (pups), and average duration of gestation observed in NPD and HFD-fed rats (Figures 1(i)–1(k)). The possible transgenerational influence of HFD feeding by parents on the prostate of weaning pups was examined, and, apart from change in the phenotypical and biochemical features such as body weight, plasma glucose level, plasma triglyceride level, plasma cholesterol level, and plasma insulin level (Fig. S3, Figure 4(d)), mild impairment in the glucose tolerance, a feature of insulin resistance, was observed in the weaning pups of HFD-fed parents as compared to that of the NPD-fed parents (Figures 4(b) and 4(c)). A significant increase in the COX-2 and p-NF-kB level and marginal increase in the p53 expression was observed in the prostate of the weaning pups of HFD-fed parents as compared to that of NPD-fed parents (Figures 4(e)–4(g)). However, no appreciable change in the expression of cell proliferation marker PCNA was observed in the weaning pups of HFD-fed parents as compared to that of NPD-fed parents (Figure 4(e)).

#### 3.3.2. Adult Pups

To examine the subsequent effects on the VP at later stage of life, a subgroup of pups was allowed to grow and was kept on different diets (either HFD or NPD) (Figure 4(a)). Biochemical analysis indicated increased plasma glucose, triglyceride, total-cholesterol, insulin, and decreased HDL-cholesterol level in the HFD-fed pups of HFD-fed parents as compared to that of NPD-fed parents (Figure 4(h)). Although all these parameters were found to be significantly less in the NPD-fed pups of HFD-fed parents as compared to HFD-fed pups of HFD-fed parents, the plasma glucose and insulin level in the NPD-fed pups of HFD-fed parents (H-NPD) were significantly higher as compared to NPD-fed pups of NPD-fed parents (Figure 4(h)). The HFD-fed adult pups (16 weeks old) of HFD-fed parents were glucose intolerant, while NPD-fed pups of HFD-fed parents (H-NPD) were not (Figure 4(i)). Next, to assess the replicative aging, SA *β*-gal activity was examined. The SA *β*-gal activity was found to be highest in the HFD-fed pups of HFD-fed parents, followed by NPD-fed pups of HFD-fed parents (H-NPD) and NPD-fed pups of NPD-fed parents (Figure 4(j)). Further, relative increase in the activity of SA *β*-gal was quantified by RGB intensity analysis of the images of VPs. A significant increase in the intensity of green and blue and decrease in red were observed in the VPs with higher X-gal staining (Fig. S2). Since the colour developed was bluish-green, the sum of these two colours was used as a measure of the SA *β*-gal activity, and a significant increase in the intensity was observed in the HFD-fed pups of HFD-fed parents as compared to the NPD-fed pups of NPD-fed parents. Although the colour intensity of NPD-fed pups of HFD-fed parents (H-NPD) was less as compared to the HFD-fed pups of HFD-fed parents, it was significantly higher than that of NPD-fed pups of NPD-fed parents (Figures 4(j) and 4(k)). An increase in the p-NF-kB level and marginal change in the p53 expression were observed in the VP of both HFD-fed and NPD-fed pups of the HFD-fed parents as compared to that of the NPD-fed pups of NPD-fed parents (Figure 4(l)). A significant increase in the expression of COX-2 and PCNA was observed in the VP of both HFD- and NPD-fed pups of HFD-fed parents as compared to that of the NPD-fed pups of NPD-fed parents (Figures 4(l) and 4(m)). Increased level of COX-2, p-NF-kB, PCNA, and SA *β*-gal activity in both HFD and H-NPD pups as compared to NPD control indicated presence of inflammation and proliferative change in the VP. The histological sections originating from the intermediate part of the VPs were examined and increased incidence epithelial infolding was observed in both HFD and H-NPD pups as compared to NPD control (Figure 4(n)).

## 4. Discussion

Clinical/epidemiological as well as experimental studies suggest an association between metabolic syndrome and aging-related prostatic disorders [3, 17]. However, the underlying molecular mechanism remained incompletely understood. Here we report that feeding rats on a diet rich in saturated fat promotes senescence-related changes and has a similar transgenerational influence on the VP. Chronic HFD feeding is known to decrease the lifespan [18] and induce glucose intolerance, hyperinsulinaemia, and insulin resistance in rodents, a type 2 diabetes like disease [19]. Previously we reported increased cell proliferation confirmed by PCNA and Ki-67 immunostaining, enhanced alpha-adrenoceptor mediated contraction, and overall enlargement of the VP in HFD-fed rats [6]. The prostatic enlargement was associated with tissue hyperplasia as evident from histological and macromolecular (DNA, RNA, and protein content normalized to the weight) analysis in the VP of HFD-fed rats [6]. Other experimental studies suggest that chronic HFD feeding increases the expression of NADPH oxidase subunits (gp91^phox^, p22^phox^, and p47^phox^) and activation of NF-kB [20, 21] and decreases the expression of glutathione peroxidase-3 [22] in the rodent prostate. Further, increased prostatic inflammation as evidenced from NF-kB activation and infiltration of inflammatory cells was observed in HFD-fed mice [20, 21]. All of these changes predispose or are indicative of the after-effects of oxidative stress and inflammation in VP of rats kept on HFD. Oxidative stress is known to contribute to the progression of several human diseases and aging. As oxidative stress can induce DNA damage and activation of p53, leading to telomere-independent senescence [23, 24], we tested whether the prostate of HFD-fed rats shows a senescence-like phenotype. Increased SA *β*-gal activity and p53 expression (both markers of cellular senescence) suggested accelerated accumulation of senescent cells in the VP of HFD-fed rats. A higher SA *β*-gal activity has also been observed in the prostate of benign prostatic hyperplasia (BPH) patients [25, 26]. Although cellular senescence is a physiological process associated with the tumor suppression and natural aging, the senescent cells can affect behaviour of neighbouring cells and can promote the late-life diseases [27]. In general, cellular senescence is considered as a state where cell gradually loses the ability to divide, but in contrast, augmented cell proliferation is the key signature of aging-related prostatic disorders (prostatic hyperplasia and prostate cancer). Although from previous studies activation of growth signaling in the VP of HFD-fed rats was known [6, 7, 28], the phenomenon was reconfirmed by measuring the level of PCNA (Figures 2(c), 3(a), and 3(b)). Prostatic enlargement, increased cellular proliferation, and contractility were reconfirmed in different laboratories later on and similar changes were observed [29, 30], strengthening the close association between HFD-induced insulin resistance and obesity with prostatic abnormalities. Significantly, increase in the expression of* both* p53 and PCNA was observed and this was of particular interest, as p53 is concerned with cell cycle arrest while PCNA is a marker of cell proliferation. To understand this phenomenon, prostatic sections were double immunostained for p53 and PCNA (sections were first immunostained for p53 followed by PCNA). p53 expression was found to be primarily restricted to the stromal cells and that of PCNA confined mainly to the epithelial cells (Figure 2(f)). It is believed that the lifespan of prostatic stromal cells is much higher than that of epithelial cells, and it probably provides an explanation for the crucial role of stromal-epithelial cells interaction in the physiological and pathological growth of prostate [31]. A recent study identifies that HFD promotes prostatic basal-to-luminal differentiation and accelerates the initiation of prostate epithelial hyperplasia [32]. The longer lifespan of stromal cells allows them to sense and store change in the microenvironment and maybe accordingly it affects the growth characteristics of the epithelial cells [31]. These results provide that (i) chronic HFD feeding leads to accumulation of senescent cells in the stroma of VP and augments (ii) proliferation of luminal epithelial cells. Literature evidences provide that direct coculture or conditioned medium from senescent prostate fibroblasts stimulates the epithelial cell proliferation in vitro [33]. Further, stromally expressed c-Jun regulates the proliferation of prostatic epithelial cells [34]. These reports underline the existence of important interactions between different cell types in progression of the prostatic diseases. Recently Vignozzi et al. reported that fat and insulin boost BPH associated prostatic inflammation [35]. Prostatic inflammation is considered as an important factor in the pathogenesis of prostatic disorders [36]. In human BPH samples a positive association was observed between expression of p53 and Ki-67 (a marker of cellular proliferation) in COX-2 positive prostatic inflammatory atrophy lesions. Further, the increased expression of p53 was found to be related to focal infiltration of macrophages [37]. The conditioned medium from macrophages activated with lipopolysaccharides induced increased expression of HIF-1*α* in prostatic epithelial cells, and under these conditions IL-1*β*, IL-6, and TNF-*α* cytokines were found to mediate HIF-1*α* induction [38]. Although further mechanistic studies are required, these findings suggest potentially important role of inflammatory cells in the prostatic pathology. A recent study demonstrates that HFD promotes prostatic basal-to-luminal differentiation and accelerates initiation of prostate epithelial hyperplasia [32]. Although the perturbation of cellular cross-talk has long been identified as a crucial determinant in the development of different prostatic disorders, to the best of our knowledge, results of the present study offer first in vivo evidence that excessive dietary fat accelerates the aging of stromal cells and enhances the luminal epithelial cell proliferation, a hallmark of aging prostatic disorders.

Environmental factors, including dietary habits, affect several disease states and are known to have transgenerational influences [13, 14]. Sandovici et al. demonstrate that maternal diet and aging can alter epigenetic control of promotor-enhancer interaction of Hnf4a gene in rat pancreatic islet [39]. Several other studies also highlight the effect of dietary habit of the mother on the methylation pattern and alteration in the gene regulation in the offspring [40–43]. Although until now it was believed that the dietary habit of mother influences the gene regulation in offspring, seminal work of Ng et al. has recently demonstrated that the dietary habit of father could also influence the future disease susceptibility of daughters [13]. Thus we asked whether or not chronic parental HFD feeding has any effect on the cellular proliferation, inflammation, and senescence-related changes in the prostate of pups. Apart from difference in the body weight, plasma glucose level, plasma triglyceride level, and plasma cholesterol level (Fig. S3), impairment in the glucose tolerance, a feature of insulin resistance, was observed in the weaning pups of HFD-fed parents (Figure 4(b)). Further, an increase in the p53 and COX-2 expressions and activation of NF-kB were also observed in the prostate of weaning pups of HFD-fed rats (Figures 4(e)–4(g)). NF-kB has been thought to play a central role in the inflammation associated aging response [44] and reversal of aging has been observed with the blockade of NF-kB signaling [45]. Kawahara et al. reported that SIRT6 regulates organismal lifespan by attenuating the NF-kB dependent gene expression [46]. Interestingly, a recent study investigating the effect of aging on the cellular and molecular composition of prostate microenvironment reported enrichment of aged stroma with the genes involved in the NF-*κ*B signaling [47]. Although no change in PCNA level indicated an absence of cellular proliferation, prostatic inflammation in the early phase of life might have important repercussions in later stage of life (Figures 4(e)–4(g)). HFD feeding by mothers during pregnancy and lactation has been known to affect the offspring's metabolism in rats [48]. Sun et al. investigated effects of maternal HFD feeding during gestation and suckling on leptin sensitivity and obesity in pups; in their study, they observed no appreciable change in the milk fat content at postnatal day 10, while a significant increase was observed at postnatal day 21 [49]. In accordance with the previous finding Purcell et al. also observed increased fat content in the milk composition between 10 and 21 postnatal days in response to the consumption of dietary fat by mothers [50]. The subsequent exposure of the foetus to the altered systemic milieu during gestation as well as lactation may be attributed to the effects of chronic HFD feeding by mothers on the altered responses of pups. The one-week-old pups of HFD-fed parents had higher plasma insulin level as compared to the pups of NPD-fed parents which further increased at the time of weaning (Figure 4(d)). To better understand the influence of chronic HFD feeding by parents and to analyze the effect at later-life, a subgroup of pups was allowed to grow and was kept on different diets (either HFD or NPD) (Figure 4(a)). HFD-fed adult (16 weeks old) pups of HFD-fed parents displayed an increased plasma triglyceride, cholesterol, glucose insulin, and decreased HDL-cholesterol level as compared to NPD-fed pups of NPD-fed parents. The plasma glucose and insulin level of NPD-fed pups of HFD-fed parents were found to be higher as compared to the NPD-fed pups of NPD-fed parents (Figure 4(h)). The HFD-fed pups of HFD-fed parents were glucose intolerant, while NPD-fed pups of HFD-fed parents were not (Figure 4(i)). Next, to assess the replicative aging, SA *β*-gal activity was examined and, as expected, it was found to be highest in the HFD-fed pups of HFD-fed parents, followed by NPD-fed pups of HFD-fed parents and NPD-fed pups of NPD-fed parents (Figures 4(j) and 4(k)). Increased SA *β*-gal activity even in the prostate of NPD-fed pups of the HFD-fed parents was of particular interest, as it provides a direct evidence of the transgenerational effects of dietary fat on the prostatic cellular senescence. Activation of inflammatory signaling in the HFD-induced prostatic disorders has been identified [51]. In consistence with our findings Benesh et al. observed hyperproliferation and altered Pten/Akt signaling in the prostates of offspring of HFD-fed dams [52]. An increase in COX-2, p-NF-kB, and PCNA level in the prostate of both HFD- and NPD-fed pups of the HFD-fed parents as compared to that of the NPD-fed pups of NPD-fed parents (Figures 4(l) and 4(m)) indicated inflammation and proliferative changes. Taken together, results of the present study provide that chronic feeding of diet rich in saturated fat by parents leads to inflammatory, proliferative, and senescence-related changes in the prostate. The limitation of present investigation remains to discern (i) whether the transgenerational influence is linked with the dietary habit of father and/or mother and (ii), if it is linked with mother, then whether the effects are transmitted during gestation and/or lactation.

## 5. Conclusions

Increased life expectancy and rising population of elderly people will demand unlocking the real cause and better therapeutics for the aging-related disorders. Prostatic hyperplasia and prostate cancer are diseases of aging prostate and accumulating evidences support its close association with the metabolic syndrome-related disorders, including type 2 diabetes, hypertension, obesity, and insulin resistance. Metabolic syndrome is a highly prevalent condition especially in developed countries and increasingly in the developing countries with changing lifestyle and dietary pattern, which typically consists of high-fat dairy products, high sugar drinks, and processed red meat. The results of present study demonstrate that chronic HFD feeding promotes accumulation of p53 expressing stromal cells, prostatic inflammation, proliferation of epithelial cells, and senescence-related changes in the prostate, supporting the emerging concept of the association between cellular senescence and late-life diseases. Further, based on these results it may be speculated that apart from better personal health and longevity, decreased consumption of fat can be a gift to the offspring by making them less susceptible to the age-related prostatic disorders.

## Supplementary Material

Figure S1: Representative photomicrographs showing PCNA immunostaining with the first or second use of PCNA antibody in prostatic sections.
Figure S2: Red, green and blue (RGB) colour intensity per unit area of the senescence-associated *β*-galactosidase stained VP.
Figure S3: Transgenerational effects of chronic HFD-feeding on the biochemical parameters.

## References

[1] Hetzl A. C., Favaro W. J., Billis A., Ferreira U., Cagnon V. H. A. (2010). Prostatic diseases in the senescence: structural and proliferative features. *Aging Male*.

[2] Bianchi-Frias D., Vakar-Lopez F., Coleman I. M., Plymate S. R., Reed M. J., Nelson P. S. (2010). The effects of aging on the molecular and cellular composition of the prostate microenvironment. *PLoS ONE*.

[3] Hammarsten J., Peeker R. (2011). Urological aspects of the metabolic syndrome. *Nature Reviews Urology*.

[4] Cai X., Haleem R., Oram S. (2001). High fat diet increases the weight of rat ventral prostate. *Prostate*.

[5] Escobar E. L. O., Gomes-Marcondes M. C. C., Carvalho H. F. (2009). Dietary fatty acid quality affects AR and PPAR*γ* levels and prostate growth. *Prostate*.

[6] Vikram A., Jena G. B., Ramarao P. (2010). Increased cell proliferation and contractility of prostate in insulin resistant rats: linking hyperinsulinemia with benign prostate hyperplasia. *Prostate*.

[7] Vikram A., Jena G., Ramarao P. (2010). Pioglitazone attenuates prostatic enlargement in diet-induced insulin-resistant rats by altering lipid distribution and hyperinsulinaemia. *British Journal of Pharmacology*.

[8] Vikram A., Jena G., Ramarao P. (2010). Insulin-resistance and benign prostatic hyperplasia: the connection. *European Journal of Pharmacology*.

[9] Hori S., Butler E., McLoughlin J. (2011). Prostate cancer and diet: food for thought?. *BJU International*.

[10] Nandeesha H., Koner B. C., Dorairajan L. N., Sen S. K. (2006). Hyperinsulinemia and dyslipidemia in non-diabetic benign prostatic hyperplasia. *Clinica Chimica Acta*.

[11] Suzuki S., Platz E. A., Kawachi I., Willett W. C., Giovannucci E. (2002). Intakes of energy and macronutrients and the risk of benign prostatic hyperplasia. *The American Journal of Clinical Nutrition*.

[12] Rinaldi J. C., Justulin L. A., Lacorte L. M. (2013). Implications of intrauterine protein malnutrition on prostate growth, maturation and aging. *Life Sciences*.

[13] Ng S.-F., Lin R. C. Y., Laybutt D. R., Barres R., Owens J. A., Morris M. J. (2010). Chronic high-fat diet in fathers programs *β*-cell dysfunction in female rat offspring. *Nature*.

[14] Jirtle R. L., Skinner M. K. (2007). Environmental epigenomics and disease susceptibility. *Nature Reviews Genetics*.

[15] Srinivasan K., Patole P. S., Kaul C. L., Ramarao P. (2004). Reversal of glucose intolerance by by pioglitazone in high fat diet-fed rats. *Methods and Findings in Experimental and Clinical Pharmacology*.

[16] Dimri G. P., Lee X., Basile G. (1995). A biomarker that identifies senescent human cells in culture and in aging skin in vivo. *Proceedings of the National Academy of Sciences of the United States of America*.

[17] De Nunzio C., Aronson W., Freedland S. J., Giovannucci E., Parsons J. K. (2012). The correlation between metabolic syndrome and prostatic diseases. *European Urology*.

[18] Baur J. A., Pearson K. J., Price N. L. (2006). Resveratrol improves health and survival of mice on a high-calorie diet. *Nature*.

[19] Storlien L. H., James D. E., Burleigh K. M., Chisholm D. J., Kraegen E. W. (1986). Fat feeding causes widespread in vivo insulin resistance, decreased energy expenditure, and obesity in rats. *American Journal of Physiology—Endocrinology and Metabolism*.

[20] Shankar E., Vykhovanets E. V., Vykhovanets O. V. (2012). High-fat diet activates pro-inflammatory response in the prostate through association of Stat-3 and NF-*κ*B. *Prostate*.

[21] Vykhovanets E. V., Shankar E., Vykhovanets O. V., Shukla S., Gupta S. (2011). High-fat diet increases NF-*κ*B signaling in the prostate of reporter mice. *Prostate*.

[22] Sekine Y., Osei-Hwedieh D., Matsuda K. (2011). High fat diet reduces the expression of glutathione peroxidase 3 in mouse prostate. *Prostate*.

[23] Campisi J. (2005). Senescent cells, tumor suppression, and organismal aging: good citizens, bad neighbors. *Cell*.

[24] Shay J. W., Wright W. E. (2005). Senescence and immortalization: role of telomeres and telomerase. *Carcinogenesis*.

[25] Choi J., Shendrik I., Peacocke M. (2000). Expression of senescence-associated beta-galactosidase in enlarged prostates from men with benign prostatic hyperplasia. *Urology*.

[26] Castro P., Giri D., Lamb D., Ittmann M. (2003). Cellular senescence in the pathogenesis of benign prostatic hyperplasia. *Prostate*.

[27] Rodier F., Campisi J. (2011). Four faces of cellular senescence. *Journal of Cell Biology*.

[28] Vikram A., Jena G., Ramarao P. (2011). Insulin-resistance reduces botulinum neurotoxin-type A induced prostatic atrophy and apoptosis in rats. *European Journal of Pharmacology*.

[29] Ribeiro D. L., Pinto M. E., Maeda S. Y., Taboga S. R., Góes R. M. (2012). High fat-induced obesity associated with insulin-resistance increases FGF-2 content and causes stromal hyperplasia in rat ventral prostate. *Cell and Tissue Research*.

[30] Chen I.-H., Chung H.-H., Cheng J.-T., Tong Y.-C. (2013). Metabolic syndrome enhances prostate contractility and in vitro phenylephrine-induced *α*1-adrenoceptor protein expression in the fructose-fed rat. *Lower Urinary Tract Symptoms*.

[31] Tunn S., Nass R., Ekkernkamp A., Schulze H., Krieg M. (1989). Evaluation of average life span of epithelial and stromal cells of human prostate by superoxide dismutase activity. *Prostate*.

[32] Kwon O., Zhang B., Zhang L., Xin L. (2016). High fat diet promotes prostatic basal-to-luminal differentiation and accelerates initiation of prostate epithelial hyperplasia originated from basal cells. *Stem Cell Research*.

[33] Bavik C., Coleman I., Dean J. P., Knudsen B., Plymate S., Nelson P. S. (2006). The gene expression program of prostate fibroblast senescence modulates neoplastic epithelial cell proliferation through paracrine mechanisms. *Cancer Research*.

[34] Li W., Wu C.-L., Febbo P. G., Olumi A. F. (2007). Stromally expressed c-Jun regulates proliferation of prostate epithelial cells. *The American Journal of Pathology*.

[35] Vignozzi L., Gacci M., Cellai I. (2013). Fat boosts, while androgen receptor activation counteracts, BPH-associated prostate inflammation. *Prostate*.

[36] Chughtai B., Lee R., Te A., Kaplan S. (2011). Role of inflammation in benign prostatic hyperplasia. *Reviews in Urology*.

[37] Wang W., Bergh A., Damber J.-E. (2009). Increased p53 immunoreactivity in proliferative inflammatory atrophy of prostate is related to focal acute inflammation. *APMIS*.

[38] Kim H.-J., Park J.-W., Cho Y.-S. (2013). Pathogenic role of HIF-1*α* in prostate hyperplasia in the presence of chronic inflammation. *Biochimica et Biophysica Acta—Molecular Basis of Disease*.

[39] Sandovici I., Smith N. H., Nitert M. D. (2011). Maternal diet and aging alter the epigenetic control of a promoter-enhancer interaction at the *Hnf4a* gene in rat pancreatic islets. *Proceedings of the National Academy of Sciences of the United States of America*.

[40] Dudley K. J., Sloboda D. M., Connor K. L., Beltrand J., Vickers M. H. (2011). Offspring of mothers fed a high fat diet display hepatic cell cycle inhibition and associated changes in gene expression and DNA methylation. *PLoS ONE*.

[41] Krasnow S. M., Nguyen M. L. T., Marks D. L. (2011). Increased maternal fat consumption during pregnancy alters body composition in neonatal mice. *American Journal of Physiology - Endocrinology and Metabolism*.

[42] Ozanne S. E., Sandovici I., Constância M. (2011). Maternal diet, aging and diabetes meet at a chromatin loop. *Aging*.

[43] Strakovsky R. S., Zhang X., Zhou D., Pan Y.-X. (2011). Gestational high fat diet programs hepatic phosphoenolpyruvate carboxykinase gene expression and histone modification in neonatal offspring rats. *The Journal of Physiology*.

[44] Salminen A., Huuskonen J., Ojala J., Kauppinen A., Kaarniranta K., Suuronen T. (2008). Activation of innate immunity system during aging: NF-kB signaling is the molecular culprit of inflamm-aging. *Ageing Research Reviews*.

[45] Adler A. S., Kawahara T. L. A., Segal E., Chang H. Y. (2008). Reversal of aging by NFkappaB blockade. *Cell Cycle*.

[46] Kawahara T. L. A., Michishita E., Adler A. S. (2009). SIRT6 links histone H3 lysine 9 deacetylation to NF-*κ*B-dependent gene expression and organismal life span. *Cell*.

[47] Bianchi-Frias D., Vakar-Lopez F., Coleman I. M., Plymate S. R., Reed M. J., Nelson P. S. (2010). The effects of aging on the molecular and cellular composition of the prostate microenvironment. *PLoS ONE*.

[48] Guo F., Jen K.-L. C. (1995). High-fat feeding during pregnancy and lactation affects offspring metabolism in rats. *Physiology and Behavior*.

[49] Sun B., Purcell R. H., Terrillion C. E., Yan J., Moran T. H., Tamashiro K. L. K. (2012). Maternal high-fat diet during gestation or suckling differentially affects offspring leptin sensitivity and obesity. *Diabetes*.

[50] Purcell R. H., Sun B., Pass L. L., Power M. L., Moran T. H., Tamashiro K. L. K. (2011). Maternal stress and high-fat diet effect on maternal behavior, milk composition, and pup ingestive behavior. *Physiology and Behavior*.

[51] Shankar E., Bhaskaran N., MacLennan G. T., Liu G., Daneshgari F., Gupta S. (2015). Inflammatory signaling involved in high-fat diet induced prostate diseases. *Journal of Urology and Research*.

[52] Benesh E. C., Humphrey P. A., Wang Q., Moley K. H. (2013). Maternal high-fat diet induces hyperproliferation and alters Pten/Akt signaling in prostates of offspring. *Scientific Reports*.

