# Supplementary material for: Parental High-Fat Diet Promotes Inflammatory and Senescence-Related Changes in Prostate
Source: Oxid Med Cell Longev. 2017 Feb 5;2017:4962950. doi: 10.1155/2017/4962950 (PMC5316447; doi:10.1155/2017/4962950)
Supplement: Supplementary file 1 — Figure S1: Representative photomicrographs showing PCNA immunostaining with the first or second use of PCNA antibody in prostatic sections. Figure S2: Red, green and blue (RGB) colour intensity per unit area of the senescence-associated β-galactosidase stained VP. Figure S3: Transgenerational effects of chronic HFD-feeding on the biochemical parameters. [file 4962950.f1.docx]

**Supplementary Information**

**Fig. S1**


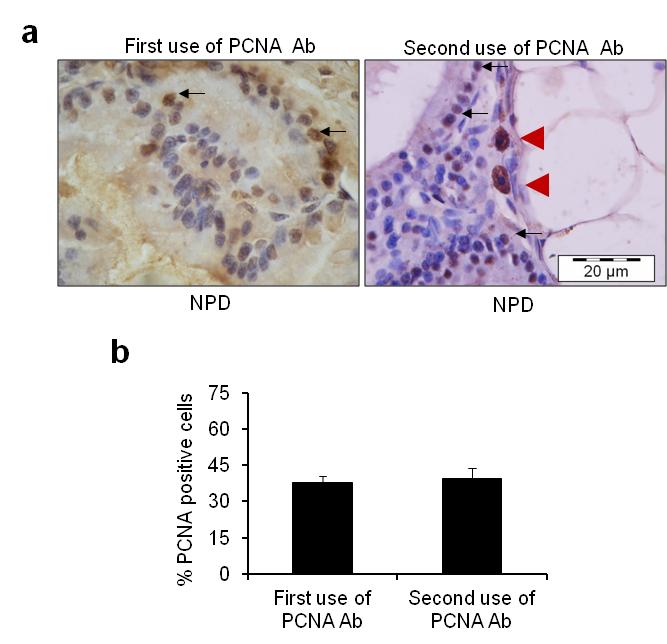


**Fig. S1.** Representative photomicrographs showing PCNA immunostaining with the first or second use (after the immunostaining of sections for p53) of PCNA antibody for the immunostaining of the prostatic sections of NPD-fed (control) rats. b) No difference in the frequency of PCNA positive cells was observed with the first or second use of PCNA antibody. PCNA positive cells are indicated by black arrows while p53 positive cells are indicated by red arrow head. All the values are shown as mean ± SEM. **P*< 0.05, *vs.* indicated group.

**Fig. S2**

**
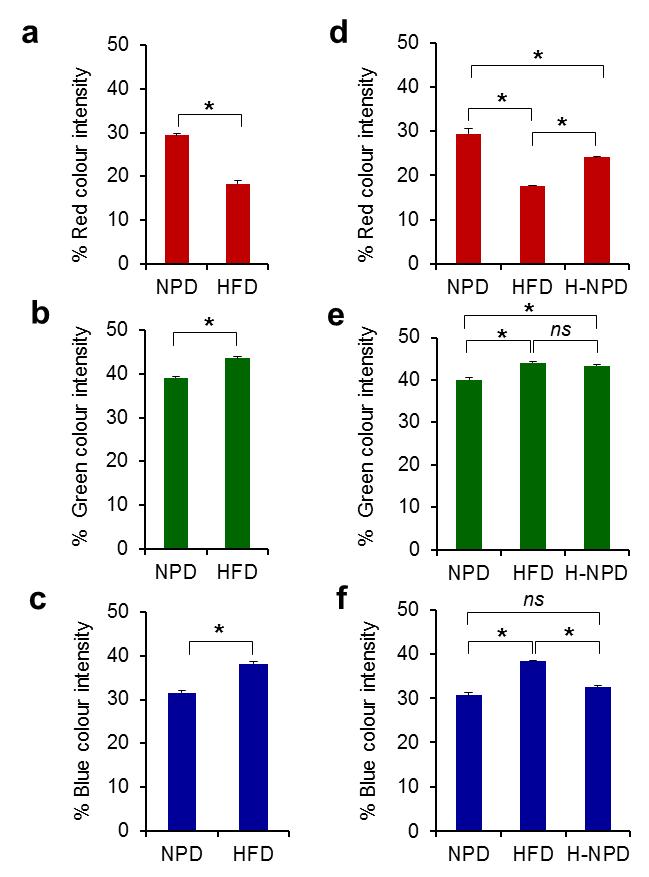
**

**Fig. S2** Red, green and blue (RGB) colour intensity per unit area of the senescence-associated β-galactosidase stained of VP. a-c) A significant increase in the intensity of green and blue colour and decrease in the intensity of red colour was observed in the VP of HFD-fed rats as compared to NPD-fed control. d-f) A significant increase in the intensity of green and blue colour and decrease in the intensity of red colour was observed in the VP of HFD-fed pups of the HFD-fed parents as compared to NPD-fed pups of NPD-fed parents. A significant increase in the intensity of green colour and decrease in the intensity of red colour was observed in the VP of NPD-fed pups of the HFD-fed parents as compared to NPD-fed pups of NPD-fed parents. All the values are shown as mean ± SEM. **P* < 0.05 *vs.* indicated group.

**Fig. S3**

**
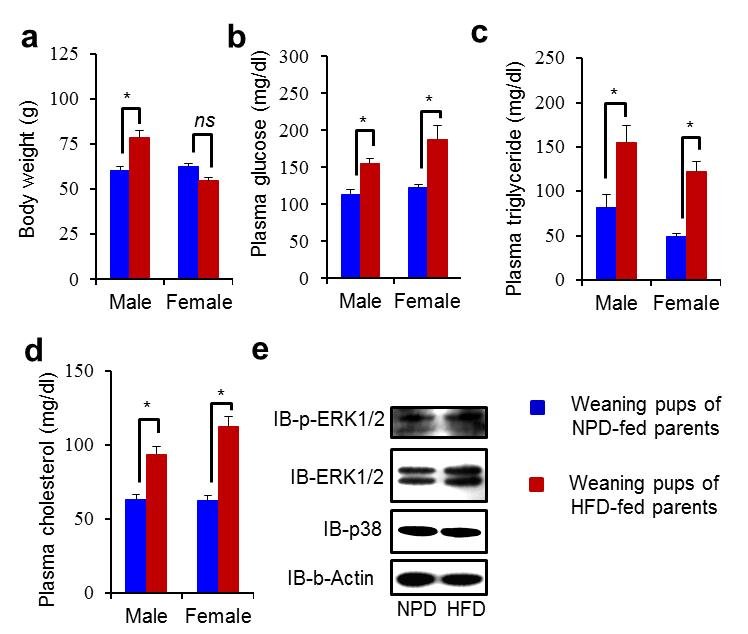
**

**Fig. S3 Trans-generational effects of chronic HFD-feeding on the biochemical parameters.** a) A significant difference in the body weight of male weaning pups of HFD-fed rats was observed as compared to that of NPD-fed control. b) A significant difference in the plasma glucose level was observed in the male as well as female weaning pups of HFD-fed rats as compared to that of NPD-fed control. c) A significant difference in the plasma triglyceride level was observed in the male as well as female weaning pups of HFD-fed rats as compared to that of NPD-fed control. d) A significant difference in the plasma cholesterol level was observed in the male as well as female weaning pups of HFD-fed rats as compared to that of NPD-fed control. e) No appreciable change in the p38 and ERK1/2 expression and phosphorylation of ERK1/2 was observed in the pups of HFD-fed rats. All the values are shown as mean ± SEM. **P*< 0.05, *vs.* indicated group.
